# Timestamp-Guided Knowledge Distillation for Robust Sensor-Based Time-Series Forecasting

**DOI:** 10.3390/s25154590

**Published:** 2025-07-24

**Authors:** Jiahe Yan, Honghui Li, Yanhui Bai, Jie Liu, Hairui Lv, Yang Bai

**Affiliations:** 1 School of Computer Science and Technology, Beijing Jiaotong University, Beijing 100044, China; 20112038@bjtu.edu.cn (J.Y.); baiyh@bjtu.edu.cn (Y.B.); jieliu@bjtu.edu.cn (J.L.); 2 Inner Mongolia High Tech Holdings Co., Ltd., Ordos 017000, China; gaokelhr@163.com (H.L.); gaokekonggu123@163.com (Y.B.)

**Keywords:** time-series forecasting, sensor data, knowledge distillation, timestamp modeling, self-distillation

## Abstract

Accurate time-series forecasting plays a vital role in sensor-driven applications such as energy monitoring, traffic flow prediction, and environmental sensing. While most existing approaches focus on extracting local patterns from historical observations, they often overlook the global temporal information embedded in timestamps. However, this information represents a valuable yet underutilized aspect of sensor-based data that can significantly enhance forecasting performance. In this paper, we propose a novel timestamp-guided knowledge distillation framework (TKDF), which integrates both historical and timestamp information through mutual learning between heterogeneous prediction branches to improve forecasting robustness. The framework comprises two complementary branches: a Backbone Model that captures local dependencies from historical sequences, and a Timestamp Mapper that learns global temporal patterns encoded in timestamp features. To enhance information transfer and reduce representational redundancy, a self-distillation mechanism is introduced within the Timestamp Mapper. Extensive experiments on multiple real-world sensor datasets—covering electricity consumption, traffic flow, and meteorological measurements—demonstrate that the TKDF consistently improves the performance of mainstream forecasting models.

## 1. Introduction

Time-series forecasting plays a vital role in various sensor-based application, including transportation [1,2,3], energy [4], and weather [5]. Since time series consist of sequential data collected at fixed time intervals, capturing both local and global dependencies is crucial for accurate prediction. Recently, neural networks have realized promising achievements owing to their strong data fitting and automatic learning capabilities. However, most of the existing studies primarily focus on local observations within historical windows, underestimating abundant global information contained in the timestamp.

Deep learning approaches, such as Recurrent Neural Networks (RNNs), Convolutional Neural Networks (CNNs), and Transformers, have been extensively validated for their capability to automatically capture temporal dependencies [6,7,8]. Nevertheless, most of existing methods adopt a sequence-to-sequence prediction paradigm, where future sequences are predicted based on continuous historical sequences. These works typically focus on historical observations within local predefined windows, while overlooking the significant role of timestamps in forecasting. In fact, the timestamp contains substantial global and seasonal information, especially in sensor-generated time series where periodicity and external environmental patterns are often prominent.

For each timestamp, temporal features such as the month, day, weekday, hour, minute, and second can be extracted, all of which are highly relevant to accurate prediction. For example, traffic volume collected from roadside sensors often differs between weekdays and weekends, as well as between peak and off-peak hours, reflecting distinct temporal patterns. Owing to their rich informational content, timestamps have the potential to provide robust global guidance for forecasting models and can even independently generate prediction outputs.

**Figure 1:**
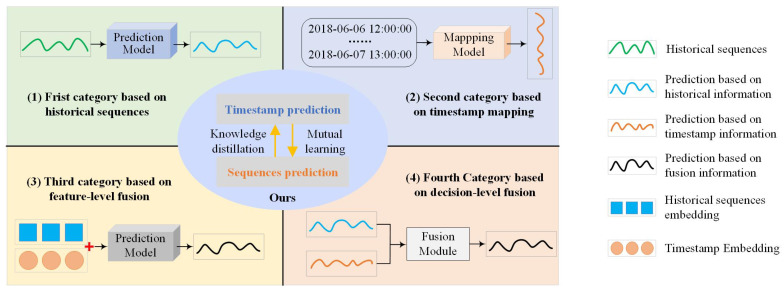
A comparison between our method and existing approaches.

Through a systematic review of state-of-the-art methods, existing works can be categorized into four groups based on how timestamps are utilized. As shown in Figure 1, the first category primarily concentrates on historical sequential observations, completely overlooking timestamps, such as Decomposition-Linear (DLinear) [9]. A significant portion of the existing works fall into this category. Instead of relying on historical sequences, the second category uses only the multiple temporal features contained in the timestamp, such as the Multi-Feature Spatial–Temporal fusion Network (MFSTN) [10]. For example, traffic volumes can be approximately estimated based on factors such as weather, holidays, day of the week, and peak hours. The third category, such as Informer [11], begins to consider timestamps by summing temporal embeddings with data embeddings. These intricate correlations prompt networks to extract information from more intuitive observations. The fourth category handles the historical sequence and timestamp independently through different models and combines two outputs using fusion weights, such as in the Global–Local Adaptive Fusion Framework (GLAFF) [12].

Despite the remarkable progress of existing models in time-series forecasting, the rich semantic information embedded in timestamps has not been fully exploited [13]. First, many approaches rely heavily on historical observations within fixed-size windows, which limits their ability to capture long-term seasonal or global patterns. Second, although timestamp features contain rich temporal semantics, they are often processed in overly simplistic ways (e.g., through summation with data embeddings), failing to unleash their potential in guiding predictions. For instance, Informer attempts to incorporate timestamp information by summing timestamp embeddings with data embeddings; the GLAFF processes the historical sequence and timestamp independently through separate models and then combines the outputs via fusion weights. However, these approaches fail to enable mutual learning between different models, which restricts their ability to fully capture the complementary knowledge within temporal data.

To overcome these limitations, we leverage knowledge distillation to facilitate the collaboration between heterogeneous models. This approach allows models to extract complementary temporal knowledge—local patterns from historical sequences and global patterns from timestamps. Through mutual guidance, these models can distill and integrate complementary information, leading to improved forecasting accuracy and robustness.

In this paper, we propose a timestamp-guided knowledge distillation framework (TKDF) that facilitates effective knowledge transfer among heterogeneous prediction models, aiming to enhance both robustness and predictive accuracy. This framework consists of a Backbone Model, which can be any sequence-based prediction model designed to capture local dependencies within a specific historical window. Additionally, a Timestamp Mapper is introduced, specifically designed to capture global dependencies by individually mapping timestamps to observation values. Unlike traditional methods, our approach integrates both local and global dependencies through mutual learning, which encourages the above two prediction branches to learn from one another, fostering a more comprehensive understanding. Notably, we incorporate self-distillation in the Timestamp Mapper to facilitate the knowledge transfer between deeper and shallower layers, thus enhancing the model’s capability. The contributions of this paper are summarized as follows:

(1) We propose a novel temporal knowledge distillation framework consisting of two prediction branches that learn from each other. The framework is highly flexibility: the Backbone Model can be any sequence-based prediction model, and the Timestamp Mapper serves as a plug-and-play component that seamlessly collaborates with the Backbone Model.
(2) We design a unique Timestamp Mapper with self-distillation. This self-distillation facilitates knowledge transfer from deeper to shallower layers within the same model, which is beneficial to enhancing its capacity to learn from broader contextual information in the timestamps.
(3) Experiments are conducted using several real-world datasets collected from sensor-based systems to demonstrate the effectiveness of the proposed model.

The subsequent sections of this paper are organized as follows: Section 2 reviews the related work on knowledge distillation and time-series prediction. Section 3 describes the overall design and details of the proposed framework. Section 4 presents the experimental results, demonstrating the effectiveness of our approach in comparison to existing methods. Section 5 concludes the paper.

## 2. Related Work

This section reviews the relevant literature about knowledge distillation, and time-series prediction, with a focus on the underlying technical principles and their associated applications.

### 2.1. Knowledge Distillation

**Figure 2:**
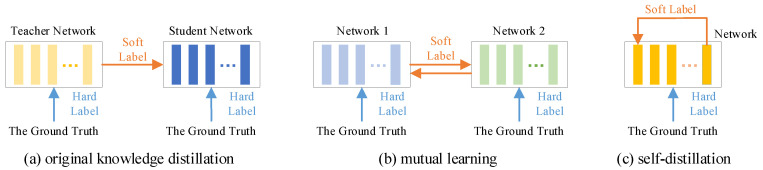
The original knowledge distillation framework and its main variations.

The original concept of knowledge distillation, proposed by Hinton et al., involves transferring knowledge from a complex teacher model with strong learning capacity to a simpler student model, thereby forming a teacher–student network [14]. The student model, typically lightweight, learns from both the ground truth (as the hard label) and the teacher’s output (as the soft label). By mimicking the behavior of the teacher through soft label supervision, the student model can achieve comparable performance while significantly reducing computational and memory overhead. Due to its superior performance, knowledge distillation has been successfully applied in the field of time-series prediction. For instance, Ma et al. proposed a multi-stage catalytic distillation framework for time-series forecasting which leverages knowledge distillation to facilitate efficient knowledge transfer in edge computing environments [15]. However, it may be challenging for a simpler student model to absorb all the knowledge from a complex teacher model. To address this issue, researchers have introduced several variations of the original knowledge distillation framework, such as mutual learning and self-distillation, as illustrated in Figure 2.

Mutual learning is a collaborative learning paradigm that enables multiple models to exchange knowledge, facilitating bidirectional interaction during the training process. This approach disrupts the traditional teacher–student relationship, allowing models to serve as both teachers and students to one another. Such interaction promotes knowledge sharing, ultimately improving prediction accuracy and enhancing the model’s generalization capability. For example, Wu et al. proposed a mutual learning module comprising multiple network branches, where interleaved multi-task supervision was employed to significantly boost performance [16]. Li et al. applied mutual learning to better capture complex correlations in traffic flow data and improve forecasting accuracy [17].

Self-distillation is a specialized form of knowledge distillation where a single network simultaneously functions as both teacher and student. Typically, the deeper layers of the network are used to supervise the shallower ones, as their outputs encode more complex and informative representations. This enables the shallower layers to acquire higher-level knowledge. Self-distillation facilitates self-optimization without the need for an external teacher model, thereby significantly reducing computational overhead and inference cost. For example, Kim et al. introduced a method called progressive self-knowledge distillation, applicable to any supervised learning task, which enhances prediction performance and generalization by distilling a model’s own knowledge [18]. Similarly, Ji et al. proposed a self-distillation mechanism during the pre-training phase, which can be regarded as an implicit regularization technique that transfers knowledge within the model itself [19].

### 2.2. Time-Series Prediction

As a significant practical challenge, time-series forecasting has attracted wide attention [20]. Time-series forecasting approaches have evolved into four categories: traditional statistics-based methods, machine learning-based methods, deep learning-based methods, and other emerging methods. The earliest methods based on traditional statistics, such as the auto-regressive integrated moving average (ARIMA) model [21], rely on temporal smoothness assumptions and are difficult to apply in practice [22,23]. Subsequently, machine learning methods, such as Support Vector Regression (SVR), became widely adopted but require large amounts of data for manual feature extraction, limiting their capacity [24]. Ensemble learning paradigms, such as bagging and boosting, have been widely adopted in time-series forecasting tasks to improve predictive performance by aggregating multiple weak learners [25]. With the development of deep learning, neural networks, including RNN-based, CNN-based, Transformer-based, and other architectures, have demonstrated satisfactory prediction performance. Among them, RNN-based methods capture temporal dependencies in time-series data dynamically through their recurrent structure [26,27,28], with examples including Long Short-Term Memory (LSTM) and Gated Recurrent Unit (GRU). Unfortunately, this architecture often suffers from the vanishing gradient problem when handling long sequences. Meanwhile, CNN-based methods rely on convolutional kernels to perform local operations [29], capturing detailed information within their limited receptive fields. This makes it difficult to capture long-term dependencies and multiple repetitive patterns using traditional convolution. To alleviate this problem, Wu et al. proposed TimesNet [30], which transforms 1D inputs into 2D space and employs 2D convolution to capture multiple periodicities. Unlike RNNs and CNNs, Transformer-based methods utilize self-attention mechanisms [31], allowing them to effectively capture long-range dependencies. However, the self-attention mechanism has quadratic complexity with respect to sequence length, making it computationally expensive for extremely long sequences. To address this, Zhou et al. proposed Informer [11], which introduces a ProbSparse self-attention mechanism that reduces both computational complexity and memory usage. As a recent variant, the iTransformer model [32] repurposes the traditional Transformer architecture by embedding time series from different channels into variate tokens which are then processed by the attention mechanism. Recently, other emerging methods have also demonstrated promising performance. Zeng et al. proposed a lightweight model, named DLinear [9], which employs only simple linear operations to accelerate both training and inference speed. With the rapid advancement of artificial intelligence, large language models (LLMs) have recently demonstrated remarkable performance in time-series forecasting [33]. Despite strong capabilities, LLMs incur high deployment and inference costs, making them less suitable for resource-constrained or low-latency prediction scenarios.

In terms of timestamps, most existing methods focus on local observations within historical windows, treating timestamps merely as auxiliary information whose global significance remains largely underestimated. For example, DLinear completely overlooks timestamps. Informer and TimesNet incorporate timestamps by adding their embeddings to position and data embeddings. iTransformer embeds timestamp features separately into variate tokens, which may compromise the physical significance of timestamps. To fully exploit timestamps, we construct heterogeneous prediction branches that independently process historical sequences and timestamps, and then integrate both local and global temporal knowledge through mutual learning to generate the final predictions.

## 3. Methods

We propose a timestamp-guided knowledge distillation framework that can efficiently integrate the local and global temporal dependencies from historical sequences and timestamp information. In time-series forecasting, given the historical sequence of c channels within h time steps $X=\{x_{1},\ldots x_{h}\}\in {\mathbb{R}}^{h\times c}$, our goal is to predict the future sequence in subsequent p time steps $Y=\{y_{1},\ldots y_{p}\}\in {\mathbb{R}}^{p\times c}$. In addition to sequential observations, we extract several timestamp features to provide global information, such as month, day, weekday, hour, minute, and second. For example, for the timestamp “2018-06-02 12:00:00” at the sampling moment t, its features can be expressed as $s_{t}=[06,02,05,12,00,00]\in {\mathbb{R}}^{1\times 6}$. Unlike observations, both the historical timestamps within the predefined window $S=\{s_{1},\ldots s_{h}\}\in {\mathbb{R}}^{h\times 6}$ and the future timestamps within the prediction window $T=\{t_{1},\ldots t_{p}\}\in {\mathbb{R}}^{p\times 6}$ are known. Therefore, we can incorporate the local observations X and global timestamps S,T to provide robustness prediction for the values of Y.

### 3.1. Overview

**Figure 3:**
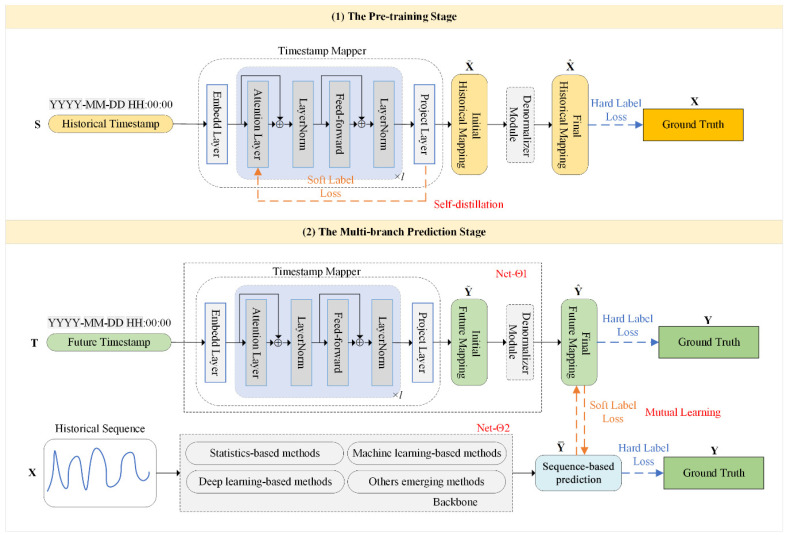
An overview of the proposed timestamp-guided knowledge distillation framework.

The proposed timestamp-guided knowledge distillation framework is illustrated in Figure 3, comprising two primary components: Timestamp Mapper and Backbone Model. Our framework offers highly flexibility: the Backbone Model can be any sequence-based model that convers historical sequences into future sequences, while the Timestamp Mapper serves as a plug-and-play component that maps timestamp features to observation values.

During the pre-training stage, the capacity of the Timestamp Mapper is enhanced through self-distillation, enabling it to learn the relationship between timestamps and observations. Since both historical observations and timestamps are available, the Timestamp Mapper can transform historical timestamps $S\in {\mathbb{R}}^{h\times 6}$ into the final historical mapping $\hat{X}\in {\mathbb{R}}^{h\times c}$, aligning them with corresponding observations $X\in {\mathbb{R}}^{h\times c}$. Self-distillation feeds deep feature representations back to the shallow layers, allowing the model to acquire global temporal contextual knowledge more effectively.

During the multi-branch prediction stage, the well-trained Timestamp Mapper further maps future timestamps $T\in {\mathbb{R}}^{p\times 6}$ to the final future mapping $\hat{Y}\in {\mathbb{R}}^{p\times c}$, while the Backbone Model offers the future prediction $\bar{Y}\in {\mathbb{R}}^{p\times c}$ based on the historical sequence $X\in {\mathbb{R}}^{h\times c}$. Both prediction results are aligned with the ground truth $Y\in {\mathbb{R}}^{p\times c}$. Mutual learning facilitates two heterogeneous prediction branches to interact with each other, enabling them to exchange complementary temporal knowledge. In this way, the proposed framework integrates the local observations and global timestamps to provide accurate and robust predictions.

### 3.2. Timestamp Mapper

As depicted in Figure 3, the Timestamp Mapper utilizes the Transformer encoder structure, which consists of an embedding layer, attention blocks, and a projection layer. Initially, each timestamp is embedded to describe its properties, then passed through attention blocks for correlation calculation, and processed by a projection layer to obtain the initial mappings. The initial historical and future mappings are denoted as $\tilde{X}\in {\mathbb{R}}^{h\times c}$ and $\tilde{Y}\in {\mathbb{R}}^{p\times c}$, respectively, conforming to standard distribution. Subsequently, the Denormalizer Module separately inverse normalizes the initial mappings $\tilde{X}$ and $\tilde{Y}$ to produce the final mappings $\hat{X}$ and $\hat{Y}$, thereby alleviating the impact of data drift.

The Timestamp Mapper can sufficiently extract the global temporal information embedded in timestamps. Specifically, the procedure of transforming historical timestamps S into an initial mapping $\tilde{X}$ is succinctly outlined as follows:

(1) $$H^{0}=\mathrm{Embedding}(S)$$

(2) $$H^{i+1}=\mathrm{Attention}(H^{i}),\;i=0,\ldots ,l-1$$

(3) $$\tilde{X}=\mathrm{Projection}(H^{l})$$

where $H^{i}\in {\mathbb{R}}^{h\times d}$ denotes the intermediate representation output by the *i*-th network layer, and d represents the dimension of the intermediate output. The attention blocks are stacked in l layers to capture the high-level temporal information embedded within the timestamps. The primary computation process for the *i*-th attention block is delineated as follows:

(4) $$H^{i}=\mathrm{LayerNorm}(H^{i}+\mathrm{MSA}(H^{i},H^{i},H^{i}))$$

(5) $$H^{i+1}=\mathrm{LayerNorm}(H^{i}+\mathrm{FeedForward}(H^{i}))$$

where LayerNorm(⋅) represents the common normalization layer and FeedForward(⋅) denotes the feedforward network. MSA(Q,K,V) indicates the multi-head self-attention mechanism, where Q,K,V represent the query, key, and value matrix, respectively. To simplify implementation, both the embedding and projection layers consist of a single linear layer. Additionally, a dropout mechanism is adopted to alleviate overfitting problems and enhance the network’s generalization. Similarly, the process of transforming future timestamps T into an initial mapping $\tilde{Y}$ mirrors the aforementioned procedure, achieved by simply substituting S and $\tilde{X}$ in Equations (1)–(3) with T and $\tilde{Y}$, respectively.

To alleviate the impact of data drift, the conventional inverse normalization approach considers mean and variance to measure the distribution deviations. However, this approach is sensitive to extreme values and lacks robustness when the observations contain anomalies. Instead of relying on the mean and standard deviation, the Denormalizer Module uses median and quantile ranges to enhance the robustness of the Timestamp Mapper against anomalies. This procedure can be succinctly expressed as follows:

(6) $$\hat{X}=\frac{\tilde{X}-\tilde{\mu }}{\tilde{\sigma }}\times \sigma +\mu$$

(7) $$\hat{Y}=\frac{\tilde{Y}-\tilde{\mu }}{\tilde{\sigma }}\times \sigma +\mu$$

where $\tilde{\mu }\in {\mathbb{R}}^{1\times c}$ and $\mu \in {\mathbb{R}}^{1\times c}$ denote the median of the initial mapping $\tilde{X}$ and the actual observation X, respectively. Similarly, $\tilde{\sigma }\in {\mathbb{R}}^{1\times c}$ and $\sigma \in {\mathbb{R}}^{1\times c}$ represent the quantile range (the distance between the q quantile and the 1−q quantile) of the initial mapping $\tilde{X}$ and the actual observation X. When q=0.75, the quantile range corresponds to the interquartile range (IQR3). This process not only helps alleviate the problem of data drift, but also makes the distribution of the final mappings more consistent with the actual observations.

### 3.3. Self-Distillation Phase

As shown in Figure 3, self-distillation is incorporated into the Timestamp Mapper during the pre-training stage, enabling it to acquire higher-level contextual knowledge more effectively. According to the relevant definitions, the knowledge distillation loss function consists of hard label loss and soft label loss. This self-distillation process can be represented as follows:

(8) $$L_{s}=\alpha L_{s}^{\left(H\right)}+(1-\alpha )L_{s}^{\left(S\right)}$$

where α is the weight hyperparameter, $L_{s}^{\left(H\right)}$ and $L_{s}^{\left(S\right)}$, respectively, represent the hard label loss and soft label loss of self-distillation. During the pre-training stage, the Timestamp Mapper maps historical timestamps S to initial historical mappings $\tilde{X}$, which are then transformed into final historical mappings $\hat{X}$ through inverse normalization, followed by alignment with the corresponding observations X. Therefore, hard label loss can be expressed as follows:

(9) $$L_{s}^{\left(H\right)}=dist(\hat{X},X)={\left\|\hat{X},X\right\|}^{2}$$

where dist(⋅) is the distance function, which is used to measure the distance between the final historical mappings $\hat{X}$ and actual observations X. Additionally, the soft label loss can be expressed as follows:

(10) $$L_{s}^{\left(S\right)}=dist(H^{0},\tilde{X})={\left\|H^{0},\tilde{X}\right\|}^{2}$$

where $H^{0}$ and $\tilde{X}$ represent the first and last layer of the Timestamp Mapper, respectively. In this way, the deep feature representations are fed back to the shallow layers, allowing the attention blocks to focus on more crucial information. The total self-distillation loss function is as follows:

(11) $$L_{s}={\left\|\hat{X},X\right\|}^{2}+{\left\|H^{0},\tilde{X}\right\|}^{2}$$

### 3.4. Mutual Learning Phase

Mutual learning involves two student networks learning from each other, as illustrated in the multi-branch prediction stage in Figure 3. Two neural networks, Net-Θ1 and Net-Θ2, have both hard label loss and soft label loss. As shown in Figure 3, the Timestamp Mapper is denoted as Net-Θ1, whose mutual learning loss function can be expressed as follows:

(12) $$L_{\theta 1}=\beta L_{\theta 1}^{\left(H\right)}+(1-\beta )L_{\theta 1}^{\left(S\right)}$$

where β is the weight hyperparameter. $L_{\theta 1}^{\left(H\right)}$ and $L_{\theta 1}^{\left(S\right)}$, respectively, represent the hard label loss and soft label loss of Net-Θ1, whose calculation process is as follows:

(13) $$L_{\theta 1}^{\left(H\right)}=dist(\hat{Y},Y)={\left\|\hat{Y},Y\right\|}^{2}$$

(14) $$L_{\theta 1}^{\left(S\right)}=dist(\hat{Y},\bar{Y})={\left\|\hat{Y},\bar{Y}\right\|}^{2}$$

where $L_{\theta 1}^{\left(H\right)}$ is used to measure the distance between the final future mapping $\hat{Y}$ and the ground truth Y, and $L_{\theta 1}^{\left(S\right)}$ is used to measure the distance between the final future mapping $\hat{Y}$ and the sequence-based prediction $\bar{Y}$. Similarly, the Backbone Model is denoted as Net-Θ2, whose mutual learning loss function is as follows:

(15) $$L_{\theta 2}=\beta L_{\theta 2}^{\left(H\right)}+(1-\beta )L_{\theta 2}^{\left(S\right)}$$

where $L_{\theta 2}^{\left(H\right)}$ and $L_{\theta 2}^{\left(S\right)}$, respectively, represent the hard label loss and soft label loss of Net-Θ2, whose calculation process is as follows:

(16) $$L_{\theta 2}^{\left(H\right)}=dist(\bar{Y},Y)={\left\|\bar{Y},Y\right\|}^{2}$$

(17) $$L_{\theta 2}^{\left(S\right)}=dist(\bar{Y},\hat{Y})={\left\|\bar{Y},\hat{Y}\right\|}^{2}$$

where $L_{\theta 2}^{\left(H\right)}$ is used to measure the sequence-based prediction $\bar{Y}$ and the ground truth Y, and $L_{\theta 2}^{\left(S\right)}$ is used to measure the distance between the sequence-based prediction $\bar{Y}$ and the final future mapping $\hat{Y}$. Mutual guidance between heterogeneous branches allows them to distill and integrate complementary knowledge, thereby improving prediction accuracy and robustness.

Overall, we employ the Timestamp Mapper and Backbone Model to extract the local and global temporal knowledge and enhance the accuracy and robustness of prediction. Algorithm 1 presents the overall process of the prediction algorithm in the TKDF.
**Algorithm 1:** Prediction algorithm process in TKDF—TKDF for Time-Series Prediction**Input:** Historical timestamp S, future timestamp T, historical sequence X
**Output:** Future sequence Y
Hyperparameter: Learning rate r, weight α,β
1. Initialization: Network $\theta_{1}$ and Network $\theta_{2}$
2. [The pre-training stage]: utilize S to predict X
3. for iter<iters do:4. Calculate self-distillation loss: $L_{s}=\alpha L_{s}^{\left(H\right)}+(1-\alpha )L_{s}^{\left(S\right)}$
5. Update parameters of the timestamp mapper: $\theta_{s}=\theta_{s}+r*\frac{\partial L_{s}}{\partial \theta_{s}}$
6. end for7. [The Multi-branch Prediction Stage]: utilize T and X to predict Y
8. for iter<iters do:9. Calculate mutual learning loss of network $\theta_{1}$: $L_{\theta 1}=\beta L_{\theta 1}^{\left(H\right)}+(1-\beta )L_{\theta 1}^{\left(S\right)}$
10. Calculate mutual learning loss of network $\theta_{2}$: $L_{\theta 2}=\beta L_{\theta 2}^{\left(H\right)}+(1-\beta )L_{\theta 2}^{\left(S\right)}$
11. Update parameters of network $\theta_{1}$: $\theta_{1}=\theta_{1}+r*\frac{\partial L_{\theta 1}}{\partial \theta_{1}}$
12. Update parameters of network $\theta_{2}$: $\theta_{2}=\theta_{2}+r*\frac{\partial L_{\theta 2}}{\partial \theta_{2}}$
13. Update the prediction result14. End for

## 4. Experiments

To verify the effectiveness of the proposed framework, three types of experiments are conducted to address the following research questions:

RQ1: Compared with the existing models for time-series prediction, does the TKDF achieve improved predictive performance?

RQ2: What are the appropriate values for the key hyperparameters?

RQ3: How do the learning strategies and individual components adopted in the TKDF contribute to predictive performance?

### 4.1. Datasets

The performance of the proposed method is evaluated on seven real-world benchmark datasets collected from sensor-based systems, including Electricity, Traffic, Weather, and four ETT datasets. These datasets are publicly available and have been widely used for performance evaluation [12]. Table 1 summarizes the statistics of these datasets. The basic information of these datasets is as follows.

- Electricity comprises hourly electricity consumption data from 321 customers, collected between 2012 and 2014.
- Traffic contains hourly road occupancy rates collected by 862 sensors installed on freeways in the San Francisco Bay Area, covering the period from 2015 to 2016.
- Weather contains 21 meteorological variables recorded every 10 min at stations across Germany in 2020.
- ETT records the oil temperature and load characteristics of two power transformers from 2016 to 2018, with measurements collected at two different resolutions (15 min and 1 h) for each transformer, resulting in four datasets: ETTh1, ETTh2, ETTm1, and ETTm2.

We follow the standard segmentation protocol by chronologically dividing each dataset into training, validation, and testing sets to prevent information leakage. The segmentation ratio is set to 6:2:2. The Adam optimizer is employed to train the model. The batch size and learning rate are set to 32 and 0.001, respectively. The number of training epochs is fixed at 50. Specifically, the length of the history window is set to 96 for the Electricity, Traffic, Weather, and all four ETT datasets, while the prediction length is varied among {96, 192, 336, 720}. The number l of attention blocks in the Timestamp Mapper is set to 2, and the dropout rate p is set to 0.1. The quantile q used in the Denormalizer Module is configured to 0.75.

### 4.2. Backbone Model

To demonstrate the effectiveness of our framework, we select several existing mainstream networks to serve as the Backbone Model, including the Transformer-based Informer (2021) [11] and iTransformer (2024) [32], the linear-based DLinear (2023) [9], and the convolution-based TimesNet (2023) [30]. All aforementioned models represent previous state-of-the-art methods for time-series forecasting tasks. (1) Informer leverages the ProbSparse attention mechanism to efficiently handle exceedingly long input sequences. It also incorporates a generative decoder to alleviate error accumulation inherent in autoregressive forecasting methods. (2) DLinear employs decomposition-enhanced linear networks to achieve competitive forecasting performance. (3) TimesNet captures two-dimensional dependencies by transforming one-dimensional time series into a collection of two-dimensional tensors. It leverages multiple periodicities to embed intra-periodic and inter-periodic variations along the columns and rows of each tensor, respectively. (4) iTransformer embeds each individual channel into a token processed by the attention mechanism, facilitating the capture of inter-channel multivariate correlations. A feedforward network is then applied to each token to learn nonlinear series representations.

As described in Section 2, these models encompass three different strategies to handle timestamps in forecasting techniques, namely summation (Informer, TimesNet), concatenation (iTransformer), and omission (DLinear).

### 4.3. Evaluation Metrics

The Mean Square Error (MSE) and Mean Absolute Error (MAE) are used to evaluate the prediction performance of the proposed method. The smaller the value of the metrics, the better the predictive performance of the model.

### 4.4. Experiment 1: Prediction Performance of the Proposed Framework

Aiming to answer RQ1, the TKDF is compared with various mainstream baselines across different architectures. The experimental results are shown in Table 2.

In Table 2, lower values indicate better predictive performance. The best results for each setting are highlighted in bold. Across most datasets and all prediction horizons, the TKDF significantly enhances the performance of nearly all baseline models, demonstrating strong generalization capability. This demonstrates that the TKDF effectively integrates global information from timestamps and local information from historical windows, thereby enhancing the robustness and predictive capability of mainstream forecasting models. This makes the TKDF well suited for long-range and ultra-long-range time-series forecasting.

On the Traffic dataset, the TKDF is significantly superior to TimesNet, DLinear, and Informer, with particularly obvious improvements at steps 336 and 720 (e.g., MAE decreases from 0.437 to 0.341). It is shown that the TKDF can better capture the temporal patterns in complex and dynamic traffic data. On the ETT datasets, the TKDF consistently achieves the best performance in both MSE and MAE for long-horizon predictions (e.g., horizon 720), indicating its superior ability to model long-term dependencies. On the Electricity and Weather datasets, although the performance gains are relatively modest, the TKDF still maintains a leading position. This indicates that the TKDF can provide stable advantages even on datasets characterized by strong periodicity and relatively stable patterns.

**Figure 4:**
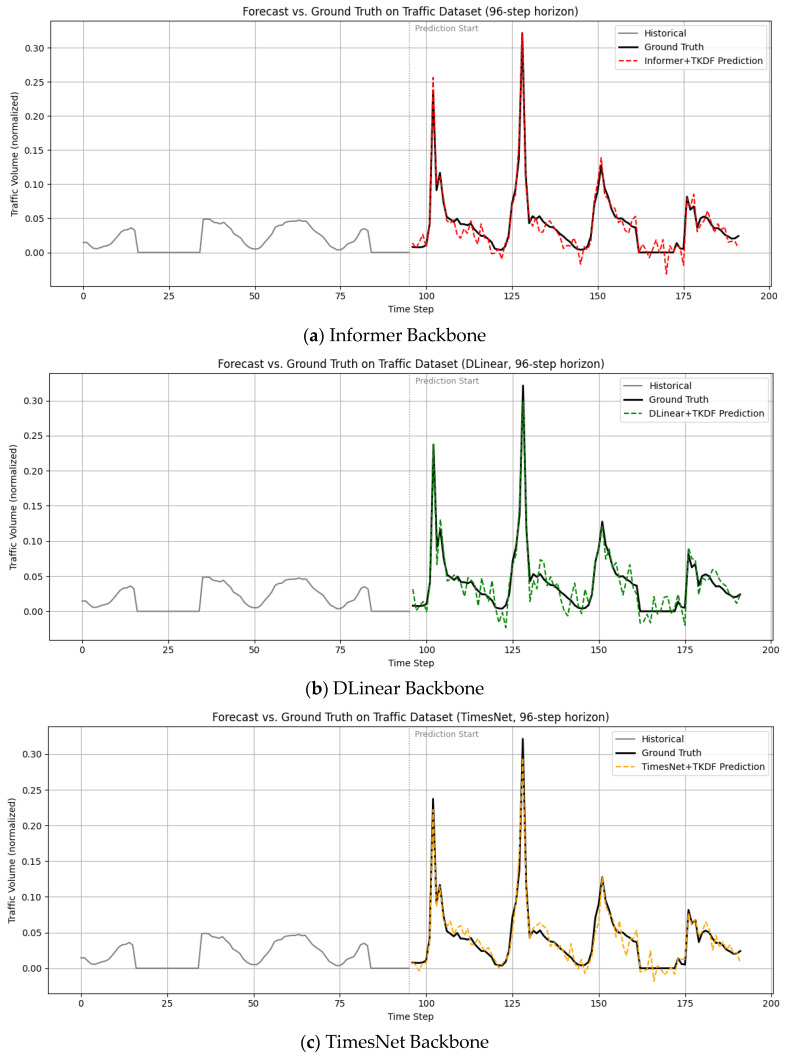

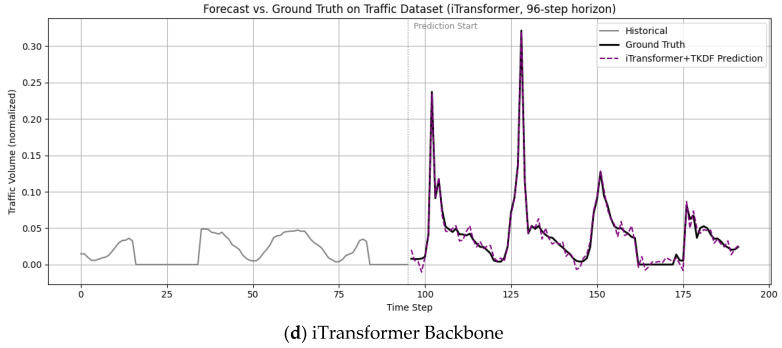
Prediction results of mainstream baseline models enhanced by TKDF.

Figure 4a–d illustrate the prediction performance of the four backbone networks of the TKDF. In Figure 4, historical data are marked in gray. The Traffic dataset records traffic volume in hourly granularity. We deliberately selected data with clear periodic patterns and used working-day data (marked in gray) as historical input to predict weekend traffic peaks. The results demonstrate that our method can effectively capture periodicity and provide accurate forecasting. Because the TKDF fully leverages the global information embedded in timestamps, it effectively captures temporal correlations and periodic patterns, enabling accurate prediction of both peak and off-peak values. It can be observed that iTransformer achieves the best prediction performance, demonstrating that the proposed TKDF is also effective when applied to state-of-the-art models.

### 4.5. Experiment 2: Parameter Determination

To address RQ2, we conduct a comprehensive hyperparameter analysis. It is worth noting that, in theory, the TKDF introduces the two most important core hyperparameters, the self-distillation weight α and the mutual learning weight β. To comprehensively compare the different parameter configurations, we also explore the quantile q of the Denormalizer Module, the number l of attention blocks, and the dropout proportion p in the Timestamp Mapper. Since iTransformer represents the previous state of the art in time-series forecasting, we implement our method using it as the backbone. The history window length is fixed at 96, while the prediction window length is set to 192. The experimental results are depicted below.

**Figure 5:**
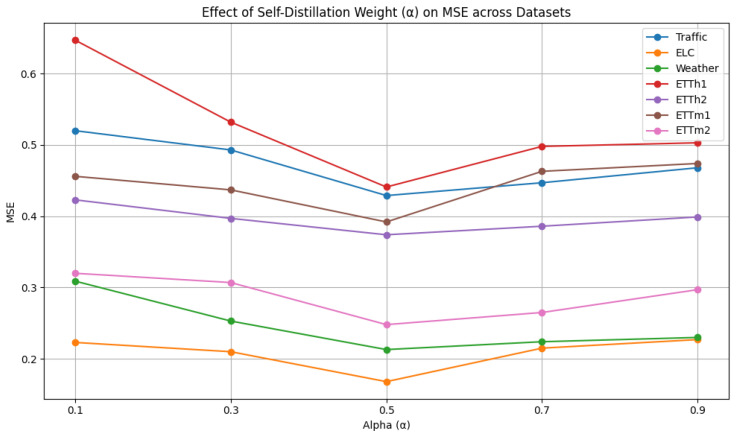
Hyperparameter analysis of self-distillation weight.

As shown in Figure 5, the self-distillation weight α is selected from {0.1, 0.3, 0.5, 0.7, 0.9}. We analyze the influence of different self-distillation weights. When α approaches 1, no soft label supervision is involved, meaning that the model relies solely on hard labels. In this case, it cannot benefit from deep semantic representations generated by the model itself and ultimately degenerates into a standard supervised learning paradigm. When α approaches 0, the model relies solely on soft labels, resulting in reduced robustness due to potential overfitting to internal representations. Therefore, the results demonstrate that moderate values (e.g., α=0.5) yield the best performance, suggesting that both soft and hard labels contribute to improved predictive performance.

**Figure 6:**
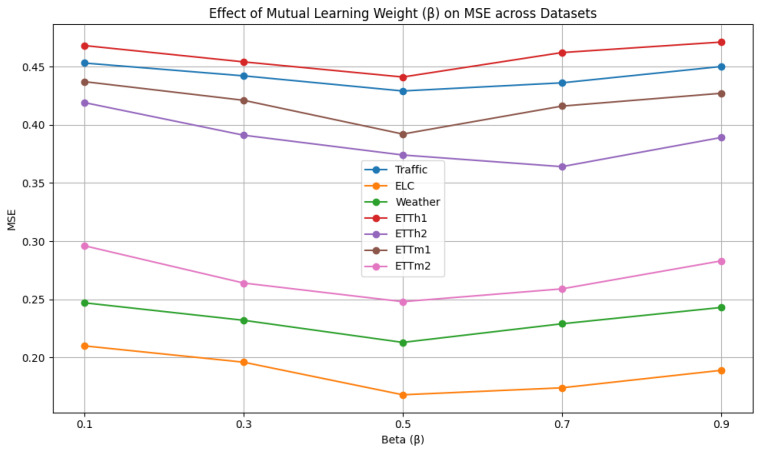
Hyperparameter analysis of mutual learning weight.

As shown in Figure 6, the mutual learning weight β is selected from {0.1, 0.3, 0.5, 0.7, 0.9}. We analyze the influence of different mutual learning weights. The model exhibits the worst performance when β=0.1 or β=0.9. A high value of β indicates that the weight assigned to the mutual learning loss is insufficient, weakening the knowledge exchange between networks and consequently degrading the prediction performance. On the contrary, a low value of β indicates that the mutual learning loss is overemphasized, while the supervision from the ground truth (hard labels) is underweighted, which significantly compromises the model’s predictive performance. When β=0.5, both networks contribute complementary information, and the model achieves its best predictive performance.

**Figure 7:**
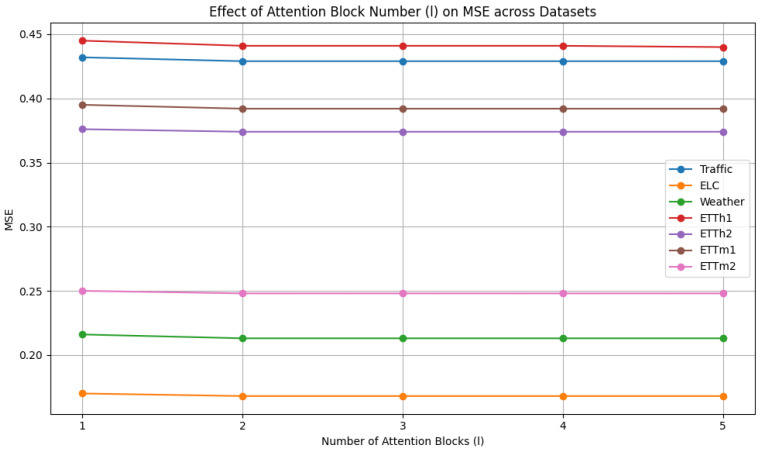
Hyperparameter analysis of attention block number.

As shown in Figure 7, the number l of attention blocks is selected from {1, 2, 3, 4, 5}. The impact of the number l of attention blocks on performance can be observed. Overall, the model exhibits low sensitivity to changes in layer depth, with two or three layers generally enough to achieve strong performance. However, increasing the network depth also leads to higher computational costs and greater difficulty in convergence. Considering training cost and model performance, we choose 2 as the number l of attention blocks.

**Figure 8:**
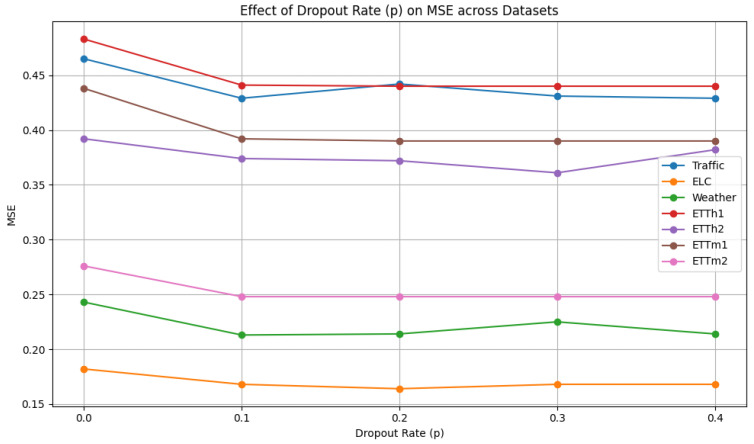
Hyperparameter analysis of dropout rate.

As shown in Figure 8, the dropout proportion p is selected from {0.0, 0.1, 0.2, 0.3, 0.4}. We can observe the change trend of dropout proportion p. Most datasets perform better at a lower dropout rate (such as 0.1 or 0.2), indicating that an excessively high dropout rate may introduce excessive information loss. To simplify the parameter selection challenge, we adopt a dropout proportion p of 0.1.

**Figure 9:**
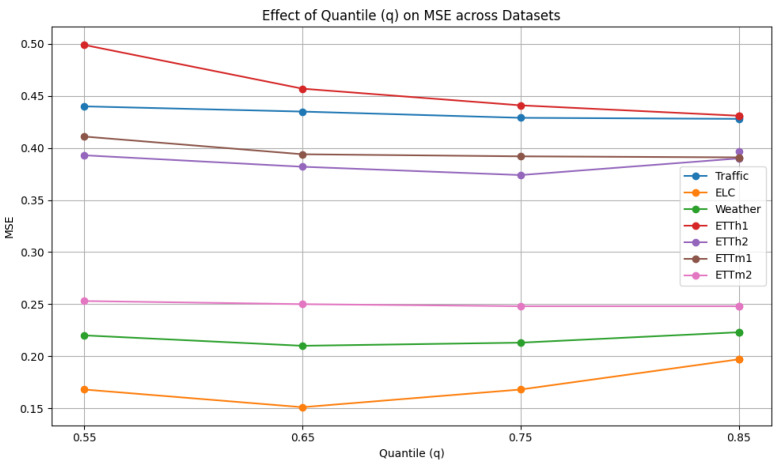
Hyperparameter analysis of quantile.

As shown Figure 9, the quantile q is selected from {0.55, 0.65, 0.75, 0.85, 0.95}. The quantile parameter in the Denormalizer Module enables the TKDF to better adapt to data drift, particularly in the presence of anomalies within the sliding window. However, higher quantile values may cause the TKDF to become overly sensitive to magnitude variations, reducing its robustness to outliers. In contrast, lower quantiles may limit the TKDF’s ability to capture distributional changes, thereby impairing its adaptability to data drift. We can observe that the performance around q=0.75 is better on almost all datasets, supporting the rationality of setting q=0.75.

### 4.6. Experiment 3: Ablation Experiments

To validate the effectiveness of the different learning strategies and core components, a series of ablation experiments are conducted below. We implement our proposed framework and its several variants using the iTransformer as the Backbone Model. The results of the ablation studies conducted on three representative benchmark datasets are summarized in Table 3.

Without self-distillation, the self-distillation mechanism within the Timestamp Mapper is removed, disabling the internal knowledge transfer between deep and shallow layers. As a result, the model exhibits a moderate performance drop across datasets, indicating that self-distillation plays a supportive role in enhancing the representation capacity of the Timestamp Mapper. This mechanism helps lower layers to incorporate richer temporal context from deeper layers, thereby improving the model’s generalization ability, especially when the timestamp information is complex or weakly periodic.

Without mutual learning, the mutual learning strategy between the Timestamp Mapper and the Backbone Model is disabled. Each component makes predictions independently without exchanging soft label guidance, equivalent to without the Backbone Model and without the Timestamp Mapper. This modification results in a noticeable degradation in performance, highlighting the importance of cross-branch collaboration. Compared to self-distillation, mutual learning leads to a more significant enhancement in predictive accuracy, as it facilitates bidirectional knowledge exchange between components, thereby capturing both local and global temporal dependencies more effectively.

Without the backbone, the Backbone Model is entirely removed from the TKDF, and predictions are made solely based on timestamps. Surprisingly, the TKDF still achieves competitive performance using only the Timestamp Mapper, highlighting its ability to effectively capture global temporal patterns embedded in the timestamps. This observation aligns with human cognitive processes when predicting traffic states, where we often do not rely on large amounts of historical data but instead make reasonably accurate predictions based on temporal features such as whether it is a weekend or peak hour. This fully demonstrates that the rich semantic information in timestamps can support independent prediction.

Without the Timestamp Mapper, the stacked attention blocks are replaced with MLP networks of the equivalent size. After this substitution, the TKDF exhibits a noticeable decline in performance, indicating that the model struggles to capture temporal dependencies among timestamps without the self-attention mechanism.

Without the quantile, the Denormalizer Module is replaced with conventional inverse normalization. The experimental results demonstrate that our proposed design is more effective. When the historical window contains anomalies, conventional normalization methods often produce inaccurate distribution estimates.

## 5. Discussion

The proposed timestamp-guided knowledge distillation framework (TKDF) has demonstrated significant improvements in forecasting robustness and accuracy across various sensor-based time-series datasets. In this section, we discuss the strengths and potential limitations of the framework, highlight its differences from existing models, and analyze its computational complexity.

### 5.1. Strengths and Innovations

The TKDF introduces a dual-branch architecture with a Backbone Model for capturing local dependencies and a Timestamp Mapper for modeling global temporal semantics. The mutual learning mechanism between these two branches enables effective integration of complementary information. Furthermore, the incorporation of self-distillation in the Timestamp Mapper allows for enhanced extraction of timestamp semantics, improving the generalization capability across different datasets.

### 5.2. Comparison with Existing Models

Unlike the GLAFF, which treats historical sequences and timestamp features independently and fuses their outputs with fixed weights, the TKDF allows for collaborative knowledge exchange between the two branches during training. This design facilitates deeper integration of local and global temporal patterns. Compared to the MFSTN, which directly maps timestamp features to predictions and neglects historical sequences, the TKDF leverages both components synergistically, ensuring richer temporal context awareness.

### 5.3. Computational Complexity Analysis

The TKDF is designed to balance accuracy and efficiency. By limiting the number of attention blocks to two and adopting lightweight components for both the Backbone Model and Timestamp Mapper, the framework maintains a manageable parameter size and training time. Preliminary observations indicate that increasing attention depth beyond two provides no noticeable improvement in accuracy while introducing additional computational overhead. This trade-off was a key consideration in finalizing the architecture.

### 5.4. Limitations and Future Work

One limitation of the TKDF is its reliance on pre-training for the Timestamp Mapper, which could increase the overall training time in large-scale applications. Additionally, while the TKDF has shown robustness across multiple datasets, its performance on highly irregular or sparse time-series data requires further investigation. Future work will explore alternative fusion strategies, adaptive attention mechanisms, and more efficient distillation techniques to address these challenges.

## 6. Conclusions

In this paper, we propose a novel timestamp-guided knowledge distillation framework (TKDF) that integrates both local and global temporal dependencies through a multi-branch mutual learning architecture. Specifically, the TKDF consists of two heterogeneous prediction branches: a Backbone Model that captures local dependencies from historical sequential data, and a Timestamp Mapper designed to model global dependencies inherent in timestamps. The two branches interact through mutual learning, allowing for bidirectional knowledge transfer via soft label supervision. Furthermore, self-distillation is incorporated into the Timestamp Mapper to enhance its capacity by transferring deep representations to shallower layers. Extensive experiments on multiple sensor-based datasets demonstrate that the TKDF significantly boosts the performance of mainstream forecasting models, particularly in capturing periodic patterns, improving robustness, and handling long-range dependencies. On the Traffic dataset, for example, the TKDF is significantly superior to TimesNet, DLinear, and Informer, with particularly obvious improvements at steps 336 and 720 (e.g., MAE decreases from 0.437 to 0.341).

## Figures and Tables

**Table 1:** The statistics of each dataset. Channel indicates the number of variates in each dataset. Length refers to the total number of time points. Frequency denotes the sampling interval.

Dataset	Channel	Length	Frequency	Information
Electricity	321	26,304	1 h	Energy
Traffic	862	17,544	1 h	Transportation
Weather	21	52,696	10 min	Climate
ETTh1 and ETTh2	7	17,420	1 h	Energy
ETTm1 and ETTm2	7	69,680	15 min	Energy

**Table 2:** The forecasting errors on multivariate datasets for the TKDF and mainstream baselines.

Models		Informer		+Ours		TimesNet		+Ours		DLinear		+Ours		iTransformer		+Ours	
Metric		MSE	MAE	MSE	MAE	MSE	MAE	MSE	MAE	MSE	MAE	MAE	MAE	MSE	MAE	MSE	MAE
Traffic	96	0.641	0.347	**0.598**	**0.332**	0.598	0.321	**0.565**	**0.306**	0.602	0.390	**0.561**	**0.368**	0.397	0.273	**0.385**	**0.265**
	192	0.708	0.398	**0.677**	**0.375**	0.623	0.339	**0.601**	**0.325**	0.637	0.398	**0.510**	**0.320**	0.438	0.281	**0.429**	**0.274**
	336	0.840	0.439	**0.702**	**0.391**	0.645	0.343	**0.627**	**0.331**	0.653	0.437	**0.514**	**0.341**	0.456	0.315	**0.437**	**0.289**
	720	0.864	0.452	**0.798**	**0.406**	0.662	0.358	**0.642**	**0.344**	0.697	0.469	**0.535**	**0.384**	0.469	0.332	**0.441**	**0.297**
Electricity	96	0.334	0.420	**0.296**	**0.383**	0.186	0.279	**0.164**	**0.245**	0.198	0.281	**0.152**	**0.242**	0.150	0.241	**0.142**	**0.231**
	192	0.365	0.434	**0.343**	**0.406**	0.203	0.298	**0.180**	**0.267**	0.204	0.290	**0.175**	**0.271**	0.169	0.257	**0.168**	**0.250**
	336	0.387	0.441	**0.369**	**0.417**	0.224	0.319	**0.192**	**0.293**	0.211	0.309	**0.194**	**0.286**	0.178	0.267	**0.170**	**0.237**
	720	0.406	0.449	**0.394**	**0.428**	0.240	0.327	**0.222**	**0.319**	0.253	0.338	**0.230**	**0.314**	0.229	0.324	**0.217**	**0.304**
ETTh1	96	0.938	0.742	**0.632**	**0.639**	0.456	0.487	**0.437**	**0.462**	0.419	0.442	**0.403**	**0.412**	0.400	0.427	**0.398**	**0.401**
	192	1.297	0.836	**0.872**	**0.698**	0.543	0.531	**0.523**	**0.513**	0.485	0.507	**0.470**	**0.483**	0.462	0.541	**0.441**	**0.528**
	336	1.368	0.878	**0.903**	**0.704**	0.621	0.572	**0.581**	**0.556**	0.526	0.523	**0.498**	**0.504**	0.503	0.458	**0.474**	**0.512**
	720	1.364	0.892	**0.973**	**0.737**	0.801	0.691	**0.704**	**0.653**	0.712	0.612	**0.697**	**0.600**	0.721	0.631	**0.698**	**0.603**
ETTh2	96	0.789	0.671	**0.768**	**0.654**	0.347	0.374	**0.296**	**0.329**	0.342	0.374	**0.324**	**0.352**	0.306	0.349	**0.298**	**0.347**
	192	1.197	0.699	**1.132**	**0.637**	0.432	0.425	**0.367**	**0.396**	0.480	0.473	**0.453**	**0.451**	0.383	0.413	**0.374**	**0.409**
	336	1.541	1.017	**1.507**	**1.001**	0.467	0.458	**0.392**	**0.431**	0.592	0.517	**0.567**	**0.498**	0.430	0.438	**0.423**	**0.421**
	720	1.797	1.208	**1.763**	**1.107**	0.483	0.469	**0.416**	**0.402**	0.643	0.596	**0.609**	**0.507**	0.433	0.454	**0.424**	**0.443**
ETTm1	96	0.598	0.561	**0.510**	**0.483**	0.369	0.417	**0.349**	**0.381**	0.332	0.387	**0.312**	**0.350**	0.396	0.407	**0.343**	**0.372**
	192	0.621	0.582	**0.539**	**0.532**	0.442	0.438	**0.411**	**0.394**	0.392	0.392	**0.367**	**0.383**	0.418	0.433	**0.392**	**0.403**
	336	0.892	0.781	**0.724**	**0.639**	0.503	0.499	**0.482**	**0.463**	0.449	0.436	**0.438**	**0.424**	0.472	0.476	**0.439**	**0.441**
	720	1.083	0.794	**0.928**	**0.732**	0.551	0.527	**0.513**	**0.513**	0.501	0.483	**0.493**	**0.487**	0.523	0.516	**0.503**	**0.498**
ETTm2	96	0.237	0.312	**0.218**	**0.270**	0.183	0.267	**0.161**	**0.232**	0.197	0.298	**0.190**	**0.293**	0.189	0.268	**0.179**	**0.263**
	192	0.314	0.347	**0.297**	**0.323**	0.240	0.313	**0.227**	**0.268**	0.293	0.371	**0.287**	**0.369**	0.264	0.318	**0.248**	**0.309**
	336	0.478	0.482	**0.463**	**0.384**	0.317	0.335	**0.304**	**0.297**	0.375	0.435	**0.363**	**0.427**	0.320	0.364	**0.307**	**0.342**
	720	0.878	0.673	**0.732**	**0.591**	0.414	0.398	**0.403**	**0.372**	0.568	0.538	**0.550**	**0.528**	0.423	0.427	**0.401**	**0.413**
Weather	96	1.248	0.867	**0.649**	**0.567**	0.178	0.231	**0.174**	**0.224**	0.198	0.257	**0.172**	**0.247**	0.180	0.225	**0.161**	**0.220**
	192	1.272	0.882	**0.872**	**0.678**	0.223	0.279	**0.221**	**0.279**	0.242	0.231	**0.224**	**0.229**	0.228	0.263	**0.213**	**0.261**
	336	1.306	1.012	**0.998**	**0.861**	0.289	0.313	**0.286**	**0.297**	0.391	0.339	**0.369**	**0.317**	0.285	0.313	**0.275**	**0.308**
	720	1.363	1.131	**1.320**	**0.892**	0.368	0.369	**0.352**	**0.362**	0.350	0.386	**0.334**	**0.365**	0.361	0.350	**0.350**	**0.346**

**Table 3:** Ablation study on TKDF and its variants.

Method		iTransformer		+Ours		w/o Mutual Learning				w/o Quantile		w/o Self-Distillation	
						w/o Backbone		w/o Timestamp Mapper					
Metric		MSE	MAE	MSE	MAE	MSE	MAE	MSE	MAE	MSE	MAE	MSE	MAE
Traffic	96	0.397	0.273	**0.385**	**0.265**	0.432	0.297	0.395	0.271	0.389	0.271	0.389	0.270
	192	0.438	0.281	**0.429**	**0.274**	0.463	0.303	0.443	0.285	0.433	0.280	0.431	0.279
	336	0.456	0.315	**0.437**	**0.289**	0.478	0.336	0.457	0.313	0.446	0.297	0.444	0.293
	720	0.469	0.332	**0.441**	**0.297**	0.510	0.351	0.470	0.336	0.459	0.322	0.451	0.310
Electricity	96	0.150	0.241	**0.142**	**0.231**	0.189	0.268	0.153	0.244	0.146	0.235	0.144	0.233
	192	0.169	0.257	**0.168**	**0.250**	0.210	0.279	0.172	0.259	0.169	0.254	0.168	0.256
	336	0.178	0.267	**0.170**	**0.237**	0.235	0.294	0.180	0.271	0.174	0.248	0.172	0.241
	720	0.229	0.324	**0.217**	**0.304**	0.267	0.348	0.231	0.330	0.223	0.317	0.220	0.310
Weather	96	0.180	0.225	**0.161**	**0.220**	0.226	0.249	0.182	0.227	0.171	0.223	0.169	0.224
	192	0.228	0.263	**0.213**	**0.261**	0.249	0.289	0.229	0.266	0.220	0.262	0.217	0.261
	336	0.285	0.313	**0.275**	**0.308**	0.317	0.343	0.287	0.315	0.279	0.311	0.279	0.308
	720	0.361	0.350	**0.350**	**0.346**	0.384	0.376	0.365	0.351	0.358	0.349	0.357	0.347

## Data Availability

Data can be provided upon request.
